# Emphysematous pyelonephritis: eight case reports and literature review

**DOI:** 10.3389/fmed.2025.1570638

**Published:** 2025-09-03

**Authors:** Du Jiang, Yang Taoyu, Wu Tao, Peng Xianyue, Gou Chenren, Liang Guobiao

**Affiliations:** ^1^Department of Urology, Affiliated Hospital of Zunyi Medical University, Zunyi, Guizhou, China; ^2^Department of Invasive Technology, Affiliated Qingyuan Hospital (Qingyuan People's Hospital), Guangzhou Medical University, Qingyuan, Guangdong, China; ^3^Department of Urology, Xinyang 154 Hospital, Xinyang, Henan, China; ^4^Department of Urology, People's Hospital of Wuchuan, Zunyi, Guizhou, China

**Keywords:** emphysematous pyelonephritis, individualized surgical treatment, surgical drainage, treatment of comorbidities, prognosis

## Abstract

**Objective:**

This study aims to discuss and summarize the diagnosis and treatment of emphysematous pyelonephritis (EPN).

**Methods:**

Retrospective medical records review of all patients diagnosed with EPN from January 2017 to June 2023 in our hospital were analyzed. A total of eight patients (three males and five females) were enrolled. The mean age was 49.38 ± 3.48 years. Among them, one case was complicated by sepsis, two by emphysematous cystitis, two by chronic renal failure, four by diabetes, and five by urolithiasis. Upon admission, all patients received aggressive antimicrobial therapy. Surgical interventions consisted of drainage procedures tailored to each patient's specific condition along with management for upper urinary tract calculi present. Clinical data were analyzed in depth and compared with the previously published studies.

**Results:**

All patients complied with the protocol of catheter removal as scheduled and were discharged after complete recovery. No recurrence of infection was recorded during the follow-up.

**Conclusions:**

Most EPN patients suffer from severe and complex medical conditions. Selecting an appropriate surgical drainage strategy can significantly reduce the rates of infection recurrence, nephrectomy and mortality, thereby improving the patient's prognosis.

## Introduction

In clinical practice, EPN is a rare event and characterized by acute necrotizing inflammation of the renal pelvis, parenchyma and surrounding tissues caused by gas-producing pathogens (1). Generally, EPN could progress rapidly and carry a high mortality rate of up to 43% if the patient has not received a timely intervention (2). Recent studies indicated lower mortality rates ranging from 0% to 37.5%, primarily attributed to advancements in sensitive antibiotic therapies and aggressive surgical approaches. Unfortunately, its rates of nephrectomy and recurrent infection remain significantly high, up to 1.4%−37.5% and 0%−28.6%, respectively (3–9). The prognosis may be influenced by the timing and efficacy of surgical treatment strategies. Hence, for those EPN patients with varying degrees of complexity, it is necessary to adopt an individualized and precise surgical approach. This can ensure adequate surgical drainage and effective management of complications, thereby significantly improving the prognosis. Here, we have retrospectively analyzed the therapeutic regimens and prognoses of eight patients with EPN in our hospital in recent years. The aim of this study is to elaborate on the diagnostic and therapeutic approaches in EPN.

## Materials and methods

A retrospective analysis was conducted on the medical records of eight patients diagnosed with EPN who were admitted to our hospital from February 2017 to June 2023. Among them, there were three males and five females, with a mean age of (49.38 ± 3.48) years. The lesions involved the left kidney in four cases and the right kidney in four cases. Comorbidities included diabetes in four cases, urolithiasis in five cases, chronic renal failure in two cases, septicemia in one cases and emphysematous cystitis in two cases. All patients exhibited symptoms of lumbalgia and fever, prompting abdominal computed tomography (CT) scans for confirmation of EPN diagnosis. Besides, their blood routine examinations revealed a total leukocyte count of (10.05 ± 2.62) × 10^9^/L, neutrophil percentage of (0.77 ± 0.03)%, platelet count of (327.9 ± 71.96) × 10^9^/L, hs-CRP of 131 ± 21.23 mg/L, hemoglobin of 95.88 ± 10.93 g/L, prealbumin of 89.75 ± 18.30 mg/L, and albumin of 27.01 ± 1.68 g/L {the classification method for EPN is presented in Table 1 [Wan. et al. (10) and Huang and Tseng (3)], and the general information of the patients is shown in Table 2}.

**Table 1:** The EPN classification based on CT imaging features.

**Classification method**	**Class and description**
Wan et al. (10)	I: Renal necrosis with the presence of gas, but no fluid II: Parenchymal gas associated with fluid in renal parenchyma, peri-nephric space, or collecting system
Huang and Tseng (3)	I: Gas in the collecting system II: Renal parenchymal gas without extension IIIA: Extension of gas into peri-nephric space IIIB: Extension of gas into pararenal space IV: EPN in solitary kidney, or bilateral disease

**Table 2:** The EPN classification, comorbidities, treatment regimens and prognosis of the patients.

**Number**	**Sex**	**Age**	**Wan grade**	**Huang grade**	**Co-morbidity**	**Bacterial culture**	**Surgical drainage**	**Treatment of comorbidities**	**Outcome**
Case 1	F	55	I	IIIA	DM/CKD	Negative	DJS	/	Well
Case 2	M	52	I	IV	CKD/staghorn calculi/solitary kidney	*E. coli*	DJS	PCNL + RIRS	Serum creatinine was maintained at 200 μmol/L
Case 3	F	51	I	IIIB	DM/ureteral calculi sepsis/thrombocytopenia/ emphysematous cystitis	Negative	DJS	/	Well
Case 4	F	40	II	IIIB	DM/rupture of right kidney/abscess of left renal kidney	*E. coli*	DJS + PCD	/	Well
Case 5	M	57	II	IIIB	Renal and ureteral calculi	*E. coli*	PCN	PCNL	Well
Case 6	F	29	II	IIIA	Renal calculi/emphysematous cystitis/nonfunctional kidney	*S. angina*	PCN	Nephrectomy	Well
Case 7	M	55	II	IIIB	DM	Negative	DJS + PCN	/	Well
Case 8	F	56	II	IIIA	Staghorn calculi	*E. faecium*	Open nephrolithotomy		Well

DM, diabetes mellitus; CKD, chronic kidney disease; PCN, percutaneous nephrostomy; PCD, percutaneous drainage; DJS, DJ stenting; E. coli, Escherichia coli; S. angina, Streptococcus angina; E. faecium, Enterococcus faecium.

**Figure 1:**
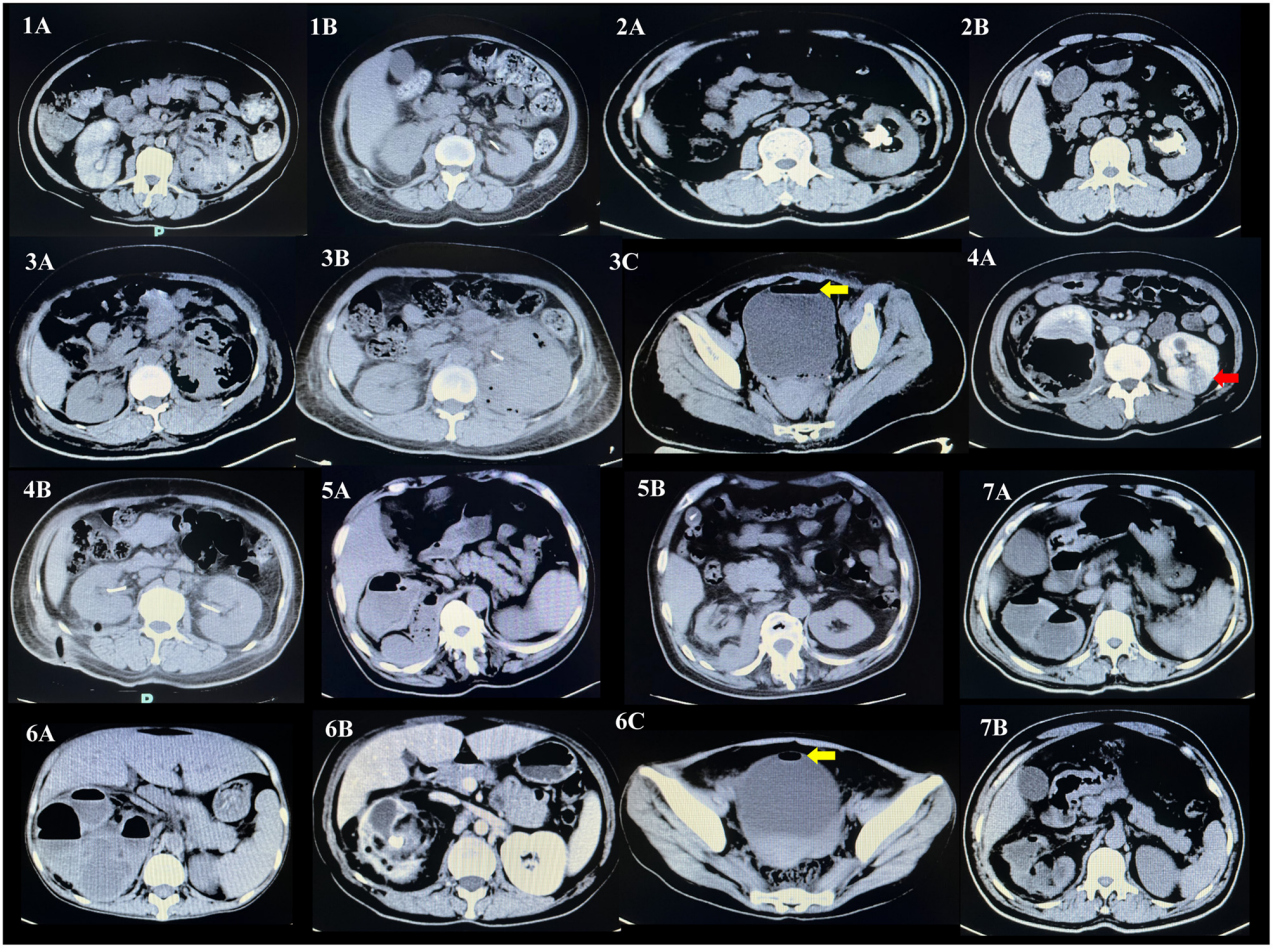
Comparative imaging data before and after operation of patient 1–7. Preoperative CT scan results: **(1A−7A)**. Recheck CT scan results 1 week after operation: **(1B−7B)**. Complicated with emphysematous cystitis: **(3C, 6C)**. The yellow arrow indicates emphysematous cystitis. Complicated with left kidney abscess: **(4A)**. The red arrow points to a renal abscess.

**Figure 2:**
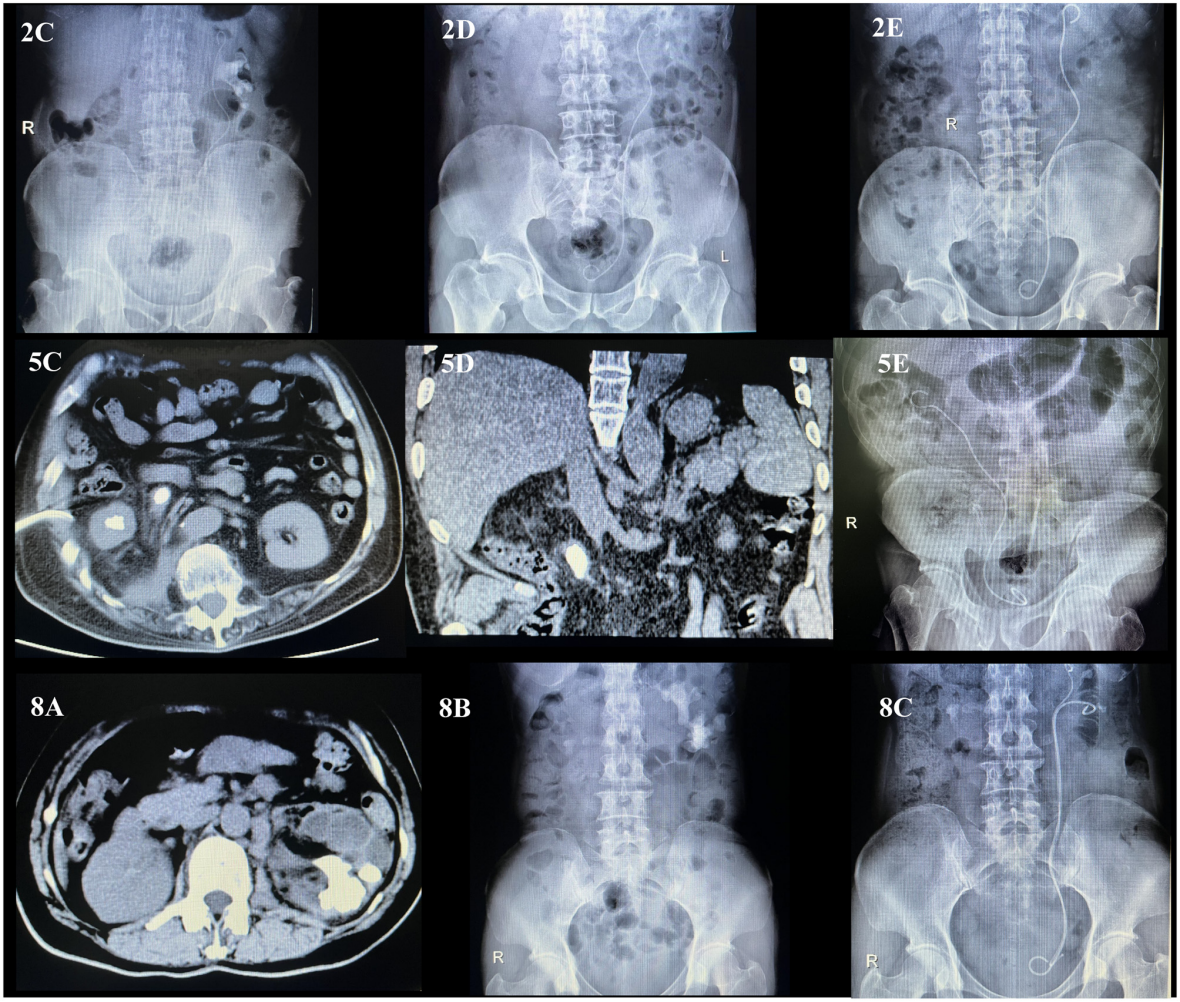
Comparative imaging data before and after operation of patient 2, 5, and 8. Preoperative CT scan results: **(2C, 5C, 5D, 8A, 8B)**. The stone-free rates after twice operation: **(2D, 2E)**. The stone-free rates after primary operation: **(5E, 8C)**.

Upon admission, all patients received broad-spectrum antibiotics for anti-infection treatment, such as piperacillin-tazobactam or imipenem-cilastatin. For patients with concurrent diabetes, controlled blood glucose was required. After comprehensive treatment, all patients improved their symptoms and were successfully discharged. During follow-up, they were in good condition and had no relapse (the specific treatment plan is shown in Table 2, the comparison of preoperative and postoperative effects is shown in Figure 1, and the comparison of fragmentation effects before and after is shown in Figure 2).

## Discussion

EPN is a relatively rare and serious infectious disease in urology, and its combination with emphysematous cystitis is even rarer (11). Its pathogenesis involves gas-producing pathogens reproduce in the renal pelvis, parenchyma, and surrounding tissue spaces, which leads to the continuous accumulation of gas and causes severe necrotizing infection. Clinically, it is more likely to occur in adult women (the ratio of men to women is 1:4 to 1:6), and there are very few reports in children (12). Risk factors contributing to its progressive disease include diabetes (up to 95%), female gender, urinary tract obstruction, and compromised immunity (1). The main pathogens associated with EPN are G– bacteria, with *Escherichia coli* being the most common isolate, followed by *Klebsiella* species and *Proteus mirabilis*. There have also been instances involving Candida and Mucormycosis pathogenicity (13–15). The microbiological characteristics of this group are basically consistent with the literature reports, with bacterial cultures of blood/urine showing. The clinical manifestations and laboratory tests in cases of EPN lack significant specificity. Consequently, its diagnosis, assessment of severity and prognosis should largely depend on CT examinations (15). With advancements in ultrasound technology, point-of-care ultrasound (POCUS) plays a critical role in rapid clinical decision-making within EPN, particularly benefiting critically ill, irremovable patients (16, 17). Remarkably, only highly sensitive C-reactive protein increased significantly on admission in our cases. In addition, one patient had a comorbid sepsis with thrombocytopenia, which led to a prolonged hospital stay.

The routine treatment of EPN is based on controlling diabetes and using sensitive antibiotics. In our study cohort, empirically selected broad spectrum antibiotics, such as piperacillin tazobactam, showed relative sensitivity to G– bacteria and covered some G+ bacteria as well. Imipenem-cilastatin was used for patients with multiple risk factors (18, 19), including advanced age, bilateral kidney involvement, shock, thrombocytopenia, and medical treatment alone. Our experience with antibiotic selection is also consistent with the study by Kai et al. (20), who concluded that antibiotics should be selected clinically in a stratified manner according to disease severity.

In the past, emergency nephrectomy was the preferred surgical treatment for EPN. By the 1980s, percutaneous nephrostomy (PCN) had begun to be used in the treatment of EPN and was widely accepted (21). Emergency nephrectomy had become the last option for serious EPN patients, and early surgical drainage could significantly reduce its rates of mortality and nephrectomy (20, 22). Currently, three surgical drainage methods are widely recognized, namely double-J stent placement (DJS), percutaneous drainage (PCD), and PCN. Nevertheless, there is still no consensus on which method is superior or should be given priority as the first option. Some researchers regarded DJS as the first option because of its good efficacy, and other advantages like lower procedural complexity, less trauma, easier care, etc. (23). Conversely, Newcomer et al. recommend PCN/PCD as the initial surgical drainage strategy (5–8). Based on our findings, the differences in these studies can be attributed to the unique advantages and disadvantages of each drainage method. Therefore, the selection of the most appropriate method should be based on the patient's specific clinical situation. For example, in Das's study, patients with type I/II underwent DJS, those with type III/IV underwent PCN, and only one patient with ureteral stones underwent open ureterolithotomy after undergoing DJS. Regardless of EPN classification, Newcomer et al. choose PCN/PCD/DJS. Therefore, different surgical drainage procedures should be selected according to patients' condition. Alternatively, combined procedures can be adopted to fully decompress and drain the infected lesions, which is more conducive to infection control. Compared with Deoraj et al. (24) who proposed choosing a treatment plan based on the classification of EPN, individualized drainage should also fully consider the patient's pathogenic factors, imaging features, and complications.

In patients with EPN, sufficient surgical drainage and rational antibiotic treatment can steadily control the infection. On this basis, effective management of complications can further improve the prognosis (25). While all patients in this study demonstrated relatively favorable outcomes, the potential for reporting bias remains given the limited sample size of this retrospective analysis. The following surgical cases are summarized to compare the advantages and disadvantages of each drainage method in different patients: ① The option of appropriate surgical drainage should be based on a comprehensive assessment of the patient's imaging findings and condition, so as to best reduce intrarenal pressure and ensure complete drainage of the infected pus. For example, in cases 1 and 3, despite the infection extending to the renal capsule or perirenal space, there was minimal gas and fluid accumulation, and no significant dilatation of the collecting system was observed. Given the presence of large amounts of gas and fluid, precise PCN puncture is challenging, and PCD also has poor drainage efficacy on separating abscess cavities. Thus, only DJS seems to be more suitable. In case 4, a single abscess due to infection unconnected to the renal pelvis required DJS in addition to PCD to relieve pressure in the renal pelvis. In some cases, like case 7, EPN patients might not get the expected results despite receiving internal drainage. At this point, an additional external drainage such as PCN is especially crucial. The poor outcome of internal drainage could be attributed to narrowing of the renal calyx neck. Therefore, those patients should choose internal and external combined drainage to achieve sufficient decompression and drainage. ② Currently, there is no consensus regarding optimal timing for surgical intervention when managing kidney stones. In this cohort, case 3 with upper urinary tract calculi and case 2 with a renal cast stone in a solitary kidney underwent staged stone removal procedures. After the first procedure combining percutaneous nephrolithotomy and lithotripsy (PCNL) with retrograde intrarenal surgery (RIRS) in case 2, the patient experienced a mild fever (38.5°C). With the infection under control, the patient underwent further RIRS to remove the ureteral stone and recovered well. Case 5 remained stable after the second stage of stone removal. Therefore, it is generally safer to perform intracavitary stone removal surgery in stages. It is also important to monitor surgical duration and maintain low-pressure irrigation. Open surgery is another relatively safe option for stone extraction that can be attempted as a one-stage procedure. In case 8 with complete staghorn calculus, one-stage open nephrolithotomy offers a reliable and safe approach for decompression, drainage, and stone clearance. It not only permits sufficient drainage of intrarenal pus and reduces the spread of infection, but also makes it possible to completely remove stones in a single procedure. Additionally, postoperative complications such as urine leakage, urinary fistula, and recurrent infections were avoided, thus sparing patients the psychological and financial burdens of multiple surgeries.

With a sample size of only eight cases (*n* = 8), it is difficult to draw generalizable conclusions. EPN is usually a complex and severe event, with considerable variation in clinical presentation across different patients. Treatment strategies for EPN should be tailored to the individual patient's condition, involving a comprehensive evaluation followed by prompt decompression and drainage. Effectively managing any comorbidities is also essential. This approach contributes to reduced infection relapse, decreased nephrectomy requirements, and lower mortality in patients.

## Data Availability

The original contributions presented in the study are included in the article/supplementary material, further inquiries can be directed to the corresponding author.
